# A single amino acid change (Y318F) in the L-arabitol dehydrogenase (LadA) from *Aspergillus niger *results in a significant increase in affinity for D-sorbitol

**DOI:** 10.1186/1471-2180-9-166

**Published:** 2009-08-12

**Authors:** Lucy Rutten, Cecile Ribot, Blanca Trejo-Aguilar, Han AB Wösten, Ronald P de Vries

**Affiliations:** 1 Department of Crystal and Structural Chemistry, Utrecht University, Padualaan 8, 3584 CH Utrecht, the Netherlands; 2 Microbiology, Department of Biology, Utrecht University, Padualaan 8, 3584 CH Utrecht, the Netherlands; 3 Functional Genomics of Plant Pathogenic Fungi UMR 5240 CNRS-UCB-INSA-Bayer CropScience Microbiology, 14-20 Rue Pierre Baizet B.P. 9163, 69263 Lyon cedex 09 France; 4 UMR BGPI, Equipe "Interactions riz-parasites", Campus International de Baillarguet, Montpellier, France; 5 Fungal Physiology, CBS Fungal Biodiversity Centre, Uppsalalaan 8, 3584 CT Utrecht, the Netherlands

## Abstract

**Background:**

L-arabitol dehydrogenase (LAD) and xylitol dehydrogenase (XDH) are involved in the degradation of L-arabinose and D-xylose, which are among the most abundant monosaccharides on earth. Previous data demonstrated that LAD and XDH not only differ in the activity on their biological substrate, but also that only XDH has significant activity on D-sorbitol and may therefore be more closely related to D-sorbitol dehydrogenases (SDH). In this study we aimed to identify residues involved in the difference in substrate specificity.

**Results:**

Phylogenetic analysis demonstrated that LAD, XDH and SDH form 3 distinct groups of the family of dehydrogenases containing an Alcohol dehydrogenase GroES-like domain (pfam08240) and likely have evolved from a common ancestor. Modelling of LadA and XdhA of the saprobic fungus *Aspergillus niger *on human SDH identified two residues in LadA (M70 and Y318), that may explain the absence of activity on D-sorbitol. While introduction of the mutation M70F in LadA of *A. niger *resulted in a nearly complete enzyme inactivation, the Y318F resulted in increased activity for L-arabitol and xylitol. Moreover, the affinity for D-sorbitol was increased in this mutant.

**Conclusion:**

These data demonstrates that Y318 of LadA contributes significantly to the substrate specificity difference between LAD and XDH/SDH.

## Background

L-arabinose and D-xylose are two of the most abundant monosaccharides in nature. They are components of the plant cell wall polysaccharides xylan, xyloglucan and pectin [1] and therefore an important carbon source for microorganisms growing on plants or plant matter. In fungi, L-arabinose and D-xylose are catabolised through the pentose catabolic pathway [2]. L-arabinose is converted to xylitol in 3 steps by the enzymes L-arabinose reductase, L-arabitol dehydrogenase and L-xylulose reductase, while D-xylose reductase converts D-xylose in a single step to xylitol. Xylitol is then converted to D-xylulose by xylitol dehydrogenase, which is subsequently phosphorylated to D-xylulose-5-phosphate that enters the pentose phosphate pathway.

The pentose catabolic pathway has been studied mainly in *Aspergillus niger*, *Aspergillus nidulans *and *Trichoderma reesei *(*Hypocrea jecorina*) and, except for L-arabinose reductase and L-xylulose reductase, all genes from the pathway have been identified and characterised [2-11]. In vitro analysis of the substrate specificity of *A. niger *L-arabitol dehydrogenase and xylitol dehydrogenase demonstrated that L-arabitol dehydrogenase is active on L-arabitol and xylitol, but not on D-sorbitol, while xylitol dehydrogenase is active on xylitol and D-sorbitol, but not on L-arabitol [5]. In this study we aimed to elucidate the structural basis for the differences in substrate specificity particularly concerning the activity on D-sorbitol.

## Results

### Fungal xylitol and L-arabitol dehydrogenases form separate groups from D-sorbitol dehydrogenases of higher eukaryotes in the family of dehydrogenases containing a Alcohol dehydrogenase GroES-like domain (pfam08240)

To determine whether fungal genomes contain homologues of D-sorbitol dehydrogenases of higher eukaryotes, the human D-sorbitol dehydrogenase [12] amino acid sequence was blasted against the genomes of *A. niger*, *A. nidulans *and *A. oryzae *at the comparative Aspergillus server from the Broad Institute http://www.broad.mit.edu/annotation/genome/aspergillus_group/MultiHome.html. However, the highest hit for these fungi was xylitol dehydrogenase (data not shown). In addition, the KEGG website http://www.genome.ad.jp/dbget-bin/www_bget?enzyme+1.1.1.15 was searched for putative D-sorbitol dehydrogenases of *A. niger*. Two of these corresponded to *ladA *and *xdhA*, while a third was An09g03900. In addition, two homologues of *A. nidulans ladA*, *ladB *and *ladC*, have been described [7] although no biochemical function has been reported for these proteins. Putative orthologues for *ladB *were only found in *A. niger *and *A. oryzae*, while orthologues for *ladC *were only absent in *N. crassa *and *T. reeseii *out of the 8 fungi tested in this study.

**Figure 1:**
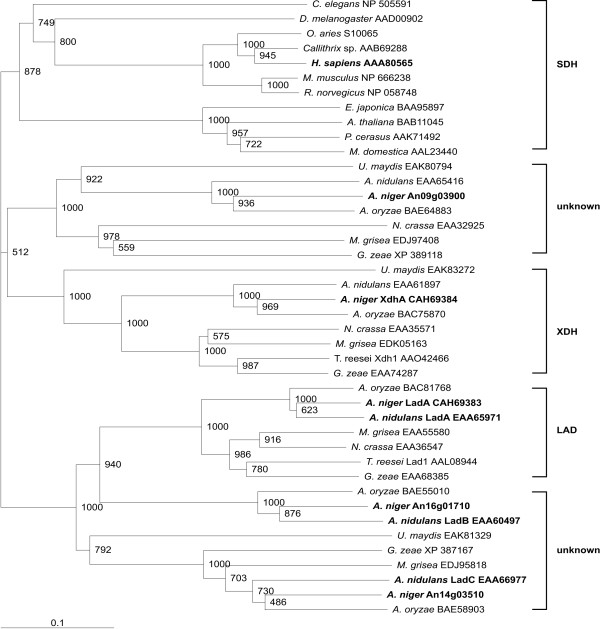
**Bootstrapped (1000 bootstraps) NJ tree of D-sorbitol, L-arabitol and xylitol dehydrogenases**. The *A. niger *enzymes, *A. nidulans *LadA, LadB and LadC and human SDH used for the modelling are in bold. Accession numbers of the protein sequences are indicated in brackets. Organisms used were 7 ascomycete fungi: *Aspergillus niger*, *Aspergillus oryzae*, *Aspergillus nidulans*, *Neurospora crassa*, *Magnaporthe grisea*, *Trichoderma reesei*, *Gibberella zeae*; 1 basidiomycete fungus:L *Ustilago maydis*; 1 nematode: *Caenorhabditis elegans*; 1 insect: *Drosophila melanogaster*; 5 mammals: *Ovis aries*, *Callithrix *sp., *Homo sapiens*, *Mus musculus*, *Rattus norvegicus*; and 4 plants: *Eriobotrya japonica*, *Arabidopsis thaliana*, *Prunus cerasus*, *Malus domestica*.

To determine the phylogenetic relationships between L-arabitol dehydrogenases, xylitol dehydrogenases and D-sorbitol dehydrogenases, an alignment was performed using amino acid sequences of established and putative L-arabitol and xylitol dehydrogenases of eight fungi, D-sorbitol dehydrogenases of ten eukaryotes and the other genes found in the analysis described above. A bootstrapped NJ tree (1000 bootstraps, Fig. 1) of the alignment shows that the D-sorbitol dehydrogenases of animals and plants split into two groups reflecting the kingdoms. The fungal L-arabitol and xylitol dehydrogenases form separate groups in the tree. In addition, a group with unknown function that contains the additional *A. niger *gene found in the KEGG database splits of from the xylitol dehydrogenase branch, although this clade only has a low bootstrap support (50%). The *ladB *and *ladC *groups split of from the *ladA *branch forming clearly defined groups.

### With respect to substrate specificity SDH and XDH are more similar to each other than either is to LAD

Previously it was reported for *A. niger *that LadA is active on L-arabitol and xylitol, but not on D-sorbitol, while XdhA is active on xylitol and D-sorbitol, but not on L-arabitol.

To determine whether D-sorbitol dehydrogenase is able to hydrolyse xylitol and L-arabitol we determined the activity of sheep liver D-sorbitol dehydrogenase on these substrates (Table 1) demonstrating that SDH has similar activity on D-sorbitol and xylitol, but significantly lower on L-arabitol.

**Table 1:** Specific activity (mmol/min/mg protein) of sheep liver SDH.

	SDH
L-arabitol	8 ± 1
Xylitol	30 ± 1
D-sorbitol	26 ± 0
Galactitol	ND
D-fructose	ND

ND = not determined.

### Modelling of the 3-dimensional structure of LadA and XdhA

**Figure 2:**
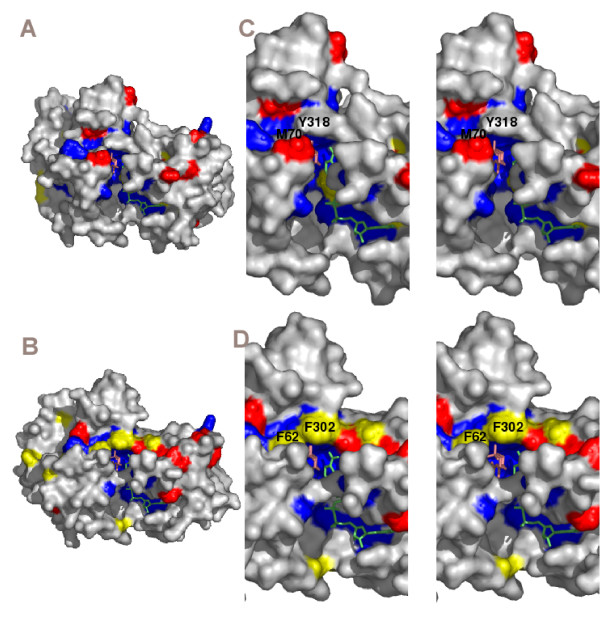
**Surface representations of theoretical models of *A. niger *LadA (A) and XdhA (B) and stereo surface representations of the active site of LadA (C) and XdhA (D)**. Amino acids that are conserved in LAD, XDH and SDH are in blue, amino acids that are only conserved in one class are in red, amino acids that are conserved in LAD and SDH (A and C) or XDH and SDH (B and D) but different in the other class are in yellow. The inhibitor and NAD are presented as sticks.

Structural models of *A. niger *LadA and XdhA were generated using the structure of human D-sorbitol dehydrogenase [12]. The position of conserved amino acids was analysed in the models. A large group of amino acids (some of which are in close proximity of the substrate) are conserved in D-sorbitol, L-arabitol and xylitol dehydrogenases (Fig. 2, in blue). In addition, both L-arabitol and xylitol dehydrogenases contain amino acids that are conserved in their own subgroup but that are different in the other dehydrogenases (Fig 2, in red). These residues are located throughout the structure. The structures have also been analysed for the location of amino acids that are conserved between L-arabitol and D-sorbitol dehydrogenases, but different in xylitol dehydrogenases (Fig 2A, in yellow). None of these amino acids are located close to the substrate. In contrast, of the amino acids that are conserved between xylitol and D-sorbitol dehydrogenases, but that are different in L-arabitol dehydrogenases, two (M70 and Y318, numbers from LadA sequence of *A. niger*) are located close to the substrate (Fig 2B, in yellow).

### Analysis of LadA M70F and Y318F

Using site directed mutagenesis, specific mutants of LadA were produced in which M70 and Y318 were altered, individually and in combination, to phenylalanine that is present at these positions in xylitol and D-sorbitol dehydrogenases. The mutant and the wild type enzymes were expressed in *E. coli *and purified. Comparison of the kinetic properties of wild type LadA and the Y318F mutant protein demonstrated that the Y318F mutant protein had a higher V_max _on L-arabitol and xylitol, but similar affinity (Km) (Table 2). In contrast, the V_max _on D-sorbitol was similar for LadA and the Y318F mutant protein, but the Km of the mutant was nearly 5-times lower (Table 2).

**Table 2:** Kinetic analysis of wild type and mutant LadA

	Wild type			Y318F		
	Km	Vmax	Kcat	Km	Vmax	Kcat

L-arabitol	0.056	96.2	863	0.078	176.8	1800
Xylitol	0.250	131.5	1180	0.218	216.8	2208
D-sorbitol	4.122	90.2	809	0.868	81.8	833

ND = not determined.

## Discussion

Comparison of the deduced amino acid sequences of LadA and XdhA to other L-arabitol, xylitol and D-sorbitol dehydrogenases, as well as some putative dehydrogenases with unknown function demonstrated that these enzymes form distinct groups in the family of dehydrogenases containing an Alcohol dehydrogenase GroES-like domain (pfam08240). Previously it was suggested that L-arabitol dehydrogenase might be the fungal orthologue of D-sorbitol dehydrogenase of higher eukaryotes [7]. However, the data in our study indicates that LAD, XDH and SDH are three distinct families, possibly originating from a common ancestor. Based on sequence identity (data not shown) and enzyme activity XDH appears to be more similar to SDH than LAD, as XDH but not LAD was shown to have significant activity on D-sorbitol [5], while SDH is significantly more active on xylitol than on L-arabitol (our study).

Interestingly, our study suggests that there is no clear fungal orthologue of SDH, based on BLAST and KEGG analysis. As the expression of *A. niger ladA *and *xdhA *appears highly specific for L-arabinose and D-xylose [5], it is unlikely that these enzymes are also acting as a sorbitol dehydrogenase for this fungus. A possible candidate sorbitol dehydrogenase might be the enzyme encoded by the uncharacterised gene from *A. niger *(An09g03900) that is in the groups that splits of the XDH branch in the tree. As orthologues for this gene were found in all tested fungi, it appears to encode a conserved function. However, bootstrap support for similarity of these enzymes to SDH is weak, indicating that no reliable prediction of function is possible based on these results.

The two homologues of LadA described for *A. nidulans *[7] cluster in the tree with LadA, but appear as separate branches. LadB appears to only be present in the Aspergilli, while LadC is present in most of the tested fungi. A previous study suggested the presence of a single L-arabitol dehydrogenase encoding gene involved in the L-arabinose catabolism [6], as a UV mutant of this gene was devoid of L-arabitol dehydrogenase activity. It is therefore likely that LadB and LadC have different biological functions f LadA.

Modelling of the structure from *A. niger *LadA and XdhA on human D-sorbitol dehydrogenase revealed a large number of amino acids that are conserved in all three types of dehydrogenases, including the residues involved in Zinc binding (H80, E81 and E166, numbers from LadA sequence) [13]. None of the residues that were conserved in L-arabitol and D-sorbitol dehydrogenases, but different in xylitol dehydrogenases were in close proximity of the substrate cleft. However, two of the residues (F62 and F302 from XdhA) that were conserved in xylitol and D-sorbitol dehydrogenases, but different in L-arabitol dehydrogenases (corresponding to M70 and Y318 from LadA) were located very close to the substrate, suggesting that they may be important for substrate specificity. As both XdhA and D-sorbitol dehydrogenase are active on D-sorbitol, whereas LadA has very little activity on this substrate [5] this could indicate that these residues are important for activity on D-sorbitol.

The M70F mutation of LadA of *A. niger *resulted in almost complete inactivation of the enzyme on a variety of substrates. The reason for this is not clear at this point, but a possible explanation could be that M70 in this particular enzyme influences the 3-dimensional structure; thus promoting enzyme activity. As the aim of this study was to identify residues important in substrate specificity, we did not further investigate this mutation.

**Figure 3:**
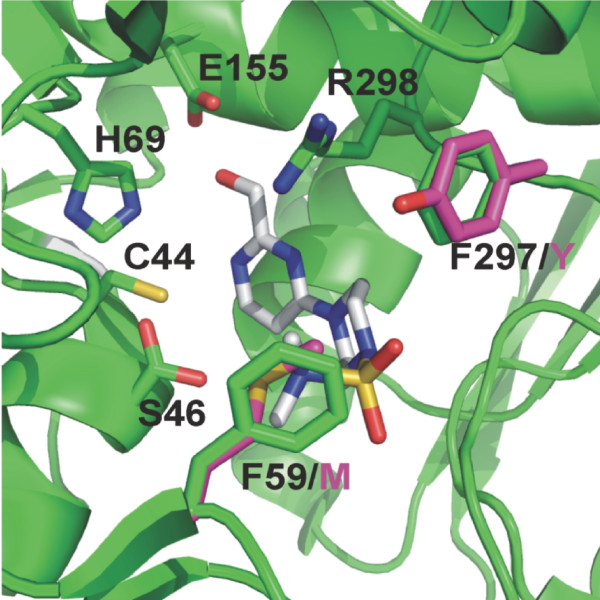
**Superposition of the active sites of D-sorbitol dehydrogenase (SDH), xylitol dehydrogenase (XDH) and L-arabitol dehydrogenase (LAD)**. Crystal structure of D-sorbitol dehydrogenase (1PL6) [12] is depicted in green. The substrate analogue which was co-crystalised is shown as grey sticks. Oxygen, nitrogen and sulphur residues are shown in red, blue and yellow, respectively. Active site residues are shown as sticks and are labelled. Residues that are different in LAD are in magenta and are labelled with the one letter code in magenta. All residues shown are identical in SDH and XDH. Numbers in the figure are from the SDH sequence: F59 corresponds to F62 and M70 in *A. niger *XdhA and LadA, respectively; F297 corresponds to F302 and Y318 in *A. niger *XdhA and LadA, respectively.

**Figure 4:**
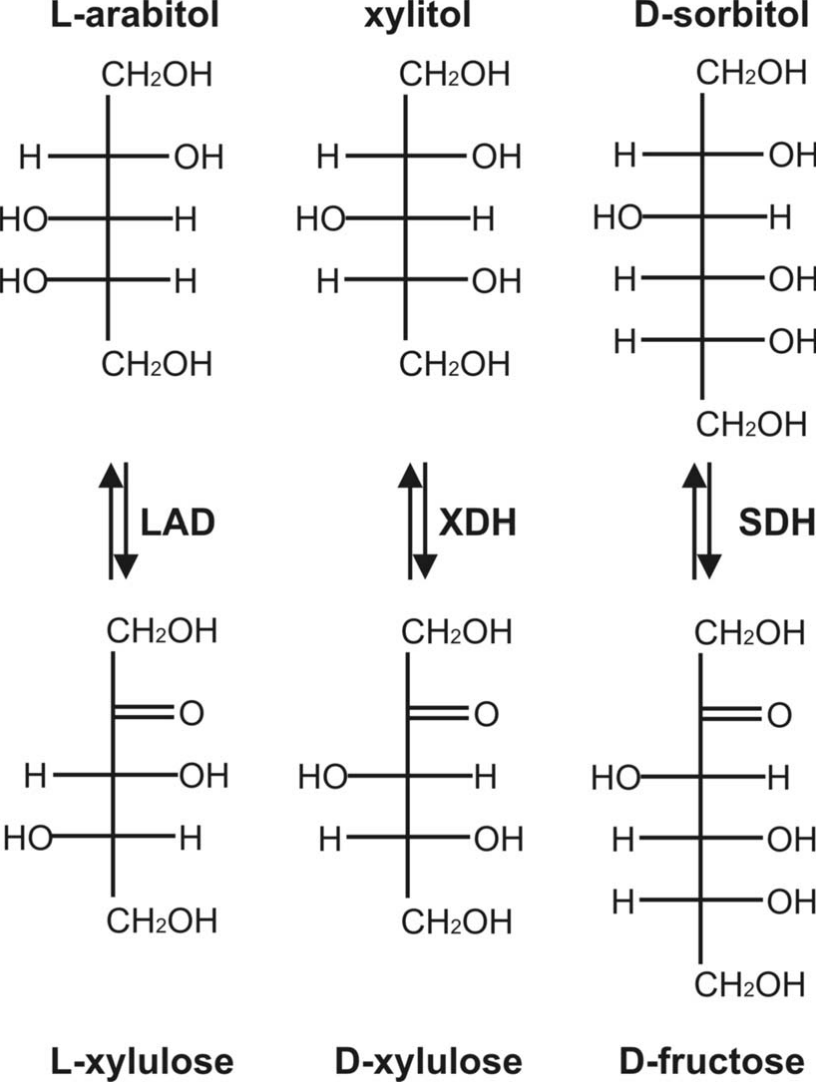
**Schematic representation of L-arabitol, xylitol and D-sorbitol and their dehydrogenase products**.

The Y318F mutation of LadA resulted in increased affinity of the enzyme for D-sorbitol, while the V_max _and K_cat _increased for L-arabitol and xylitol. Projection of the catalytic site of LAD, SDH and XDH predicts that the tyrosine residue in LAD and the phenylalanine in SDH and XDH are in exactly the same position (Fig. 3). This suggests that the OH group on the Y318 is the only structural difference between LadA and the Y318F mutant protein. This demonstrates that the presence of a phenylalanine at this position contributes significantly to D-sorbitol dehydrogenase activity. This OH-group probably affects positioning of D-sorbitol by hydrogen-bond formation in the substrate binding site, which prevents efficient catalysis in native *A. niger *LadA. The tyrosine residue does not affect affinity of LadA for L-arabitol and xylitol. However, the increased activity in the mutant suggests that the presence of the OH-group delays release of the products (L-xylulose and D-xylulose). D-sorbitol and xylitol differ structurally from L-arabitol with respect to positioning of the OH-group on C2 and C4, while D-sorbitol has an additional OH group at C5 compared to xylitol (Fig. 4). The increased affinity for D-sorbitol but not for L-arabitol or xylitol of the Y318F mutant may suggest that the presence of the OH group on Y318 in LadA interferes with the OH group on C5 of L-arabitol, resulting in a conformation for D-sorbitol in the active site that inhibits enzymatic conversion.

Genomes are continuously subjected to sequence mutations, resulting in evolution of species and biodiversity. Mutations that result in beneficial changes are likely to be maintained, while disadvantageous mutations will lose out in natural selection and therefore disappear again. The higher activity on L-arabitol of the Y318F mutant protein suggests an evolutionary advantage for this mutation with respect to conversion of this compound and therefore the efficiency of this metabolic pathway. This could indicate that this step in the pathway is not rate-limiting and therefore increased activity does not result in a biological advantage. Alternatively, since the increased activity is accompanied by a reduction in specificity this could provide selection against this mutation. It may be disadvantageous to convert other substrates simultaneously with L-arabitol, either due to competition for the enzyme or because the resulting product have a negative effect on growth.

## Conclusion

In conclusion we have shown that xylitol dehydrogenases are more closely related to D-sorbitol dehydrogenases than L-arabitol dehydrogenases. Moreover, we proved that the Y318F mutation is important for activity on D-sorbitol of L-arabitol dehydrogenase. These data increase our understanding of the molecular basis of substrate specificity of these closely related enzyme classes.

## Methods

### Strains and plasmids

*Escherichia coli *DH5αF' and M15 [pREP4] were used for routine plasmid propagation and for enzyme production, respectively. Cloning was performed using pBluescript SK^+ ^[14], pGEM-T easy (Promega) and pQE32 (Qiagen).

### Molecular biology methods

Standard methods were used for DNA manipulations, such as cloning, DNA digestion, and plasmid DNA isolation [15]. Sequence analysis was performed using the Big Dye Terminator kit, Version 1.1 (Applied Biosystems, Foster City, CA) according to the supplier's instructions. The reactions were analysed with an ABI 310 (Applied Biosystems) or on an ABI 377 (Applied Biosystems) in which case Longranger Single Packs (Cambrex Bio Science, Rockland, Inc., Rockland, ME) were used.

### Sequence analysis

Nucleotide sequences were analysed with computer programs based on those of Devereux *et al*. [16]. Sequence alignments were performed by using the Blast programs [17] at the server of the National Center for Biotechnology Information, Bethesda, Md., USA http://www.ncbi.nlm.nih.gov/blast/. Multiple sequence alignments and construction of the bootstrap tree were performed using ClustalX2.0 [18]

### Production of recombinant LadA

Derivatives of the expression vector pQE32 containing wild type and mutated versions of *ladA *were transformed to *E. coli *M13 cells (Qiagen). Transformation and purification of the recombinant proteins using Ni-agarose (Qiagen) was performed according to the supplier's instructions.

### Enzyme assays

All enzyme assays were performed at 20°C. Dehydrogenase activities were determined using 100 mM glycine pH 9.6, 0.4 mM NAD^+ ^and 100 mM substrate. Reductase activities were determined using 50 mM sodium phosphate pH 7.6, 0.2 mM NADH and 100 mM substrate. Absorbance changes at 340 nm (ε = 6.22 mM^-1 ^cm^-1^) were measured on a Unicam UV-1 spectrophotometer (Spectronic Unicam, Rochester, NY). Sheep liver SDH was obtained from Sigma (S3764).

### Modelling

Models of *A. niger *LadA and XdhA structures were generated using the SWISS-MODEL program http://swissmodel.expasy.org//SWISS-MODEL.html[19-21] with a crystal structure of D-sorbitol dehydrogenase (Protein Data Bank code: 1PL6). In this structure human D-sorbitol dehydrogenase is in complex with the cofactor NAD and an inhibitor [12]. The models were represented using the software package PYMOL [22].

### Site-directed mutagenesis

Site directed mutagenesis was performed using the Quik Change protocol (Stratagene, La Jolla, Calif.). Two complementary oligonucleotides of 30–34 nucleotides were designed for each mutation, carrying the mutation in the middle of the oligonucleotide. PCR mixtures contained 50 ng of DNA template, 125 ng of each oligonucleotide, 1 μl of a 10 mM dNTP stock, 5 μl of 10× pfu buffer, and sterile water to a total volume of 24 μl. Before the start of the PCR, 1 μl of pfu DNA polymerase (Stratagene) was added. The reaction parameters were: denaturation of the DNA for 5 min at 95°C, followed by 16 cycles of 30 s denaturation (95°C), 1 min annealing (56°C) and 15 min amplification (68°C). The product was incubated for 4 h with DpnI at 37°C. This enzyme degrades methylated (template) DNA but not the DNA amplified during the PCR.

## Authors' contributions

LR carried out the modelling studies. CR and BTA carried out the biochemical analysis. RPdV drafted the manuscript. All authors read and approved the final manuscript.
